# Understanding the Molecule-Electrode Interface for Molecular Spintronic Devices: A Computational and Experimental Study

**DOI:** 10.3390/molecules23061441

**Published:** 2018-06-13

**Authors:** Lidia Rosado Piquer, Raquel Royo Sánchez, E. Carolina Sañudo, Jorge Echeverría

**Affiliations:** 1 Departament de Química Inorgànica, Secció de Química Inorgànica; C/Martí i Franqués 1-11, 08028 Barcelona, Spain; li.rosado.p@gmail.com (L.R.P.); raquelroyo@hotmail.es (R.R.S.); 2 Institut de Nanociència i Nanotecnologia (IN2UB), C/Martí i Franqués 1-11, 08028 Barcelona, Spain; 3 Institut de Química Teòrica i Computacional (IQTC-UB), Universitat Barcelona, Martí i Franqués 1-11, 08028 Barcelona, Spain

**Keywords:** lanthanide single-molecule magnets, iron oxide nanoparticles, weak interactions

## Abstract

A triple-decker SYML-Dy2 single-molecule magnet (SMM) was synthetized and grafted onto the surface of iron oxide nanoparticles (IO-NPs) coated by an oleic acid monolayer. The magnetism of the SYML-Dy2 complex, and the hybrid system, NP-Dy2, were studied by a superconducting quantum interference device (SQUID). Density functional theory (DFT) calculations were carried out to study both the energetics of the interaction between SYML-Dy2 complex to the organic capping, and the assembly presented by the oleic acid chains.

## 1. Introduction

The use of magnetic molecules in devices has been a goal since the discovery of single-molecule magnets (SMMs) in the 1990s. SMMs can retain the magnetization and act like nanomagnets. This behavior is due to a high spin ground state (S) and the large axial magnetic anisotropy (D) of the easy axis type, with a negative zero-field splitting (ZFS) [1]. As a consequence, there is an energy barrier for the relaxation of the magnetization. This phenomenon makes such materials useful for use in high-density magnetic storage devices. Moreover, the molecular nature of the SMMs can also exhibit quantum tunneling of magnetization (QTM), and could be used as qubits in quantum computing [2,3].

The discovery of the first SMM, the dodeca-nuclear complex ($\left[{\mathrm{Mn}}_{12}O_{12}{\left({\mathrm{CH}}_{3}\mathrm{COO}\right)}_{16}{\left(H_{2}O\right)}_{4}\right]$) [1], gave rise to the design of new transition metal complexes, including a large family of Mn_12_ complexes, in order to improve the blocking temperatures and the anisotropy barrier. In 2003, it was reported that lanthanide phthalocyanine sandwich complexes, ${\left[{\mathrm{LnPc}}_{2}\right]}^{n}$ (Ln^III^ = Tb, Dy, Ho; H_2_Pc = phthalocyanine; *n* = −1, 0, +1), could display unprecedented relaxation behavior, leading to the gradual acceptance of use Ln-based SMMs [4]. For lanthanides, the interaction between the ground J state with the crystal field promotes a magnetic anisotropy barrier allowing the separation of opposite orientations of the spin ground state [5]. A bistable ground state is obtained for Dy (III) with a crystal field for which the ligand electron density is concentrated above and below the *xy* plane, like what happens in sandwich-type ligand geometry. For this study we chose to prepare the Dy analogue of our reported sandwich complexes [Ln_2_(SYML)_3_(H_2_O)] (Ln = Yb, Er) [6].

Molecular spintronics is an emerging branch in nanotechnology, and one of the most active research areas within nanomagnetism. Spintronics was born in 1988, with the Nobel Prize being awarded to Fert [7] and Grünberg [8] in 2007; it has resulted in applications in hard drive information storage and magnetic sensing. Recent advances have shown that single-molecule magnets can be successfully transferred to surfaces with retention of their magnetic behavior, and potentially exploited as spintronic devices [9]. Magnetic surfaces in the form of iron oxide nanoparticles (NPs) are used to simulate a simple break-junction electrode acting as the support for a hybrid material containing a 4f molecular nanomagnet [10,11]. Magnetite (Fe_3_O_4_) NPs are superparamagnetic, and can be prepared by high-temperature decomposition of iron precursors in an organic medium, with oleic acid as a surfactant [12]. The resulting self-assembled monolayer (SAM) of the organic acid onto the NPs would prevent further oxidation to Fe_2_O_3_, which could be vital for magnetization. Even so, the key role of this nonmagnetic organic acid capping layer consists of addressing the decoupling of the SMM to the surface [13,14]. It has been demonstrated that the magnetoresistance response of a molecular spintronic device hinges upon the interface between the ferromagnetic surface and the molecular layer. The deposition of SMMs on a naked magnetic surface affects their molecular properties by coupling with the surface; thus, a decrease of magnetization takes place. In this context, it is vital to control the chemical bonding or physisorption between the magnetic molecules and magnetic electrodes in order to tailor the exchange coupling interaction, and subsequently, the magnetoresistance response of the device. An enhancement of the magnetic properties of the iron oxide NP was observed by Prado et al. by coordination of a Co(II) coordination complex to Fe_2_O_3_ NPs [15]. They propose that covalent linking of the Co(II) complex to Fe_2_O_3_ through oxo-bridges, and the resulting magnetic interaction, are key for the observed enhancement of the magnetic properties. Our system is completely different: the SAM of oleic acid caps the iron oxide NP, and ensures that there is no direct magnetic exchange coupling between the iron oxide and the molecular SMM.

Both the conformation of the capping monolayer and the adsorption of the SMM onto it are controlled by noncovalent interactions. Therefore, the understanding of the nature and strength of such interactions is a determining factor in achieving a stable aggregate. Dispersion corrected density functional theory (DFT-D) methods, which are able to capture electron correlation, represent a non-expensive way to computationally analyze dispersion-bound systems [16]. We perform here a DFT-D study of the internal conformation of the oleic acid monolayer on a simplified model, as well as of the capping layer-SMM interface, to demonstrate that the adsorption of the SMM is energetically favorable.

## 2. Experimental

All chemicals and solvents were purchased from commercial sources and used as received.

### 2.1. C_28_H_20_N_2_O_2_ (SYMLH_2_)

SYMLH_2_ (*N*,*N*′-bis (1-naphthaldiamine)-*o*-phenylendiamine) was synthesized according to the literature methods [6]. *o*-Phenylendiamine (0.54 g, 5 mmol) is dissolved in 20 mL of ethanol. To this solution, 1.72 g (10 mmol) of 2-hydroxy-1-naphthaldehyde in 20 mL ethanol was added. The resulting solution was refluxed for 2 h. The obtained orange precipitate was filtered off and then dried with anhydrous diethyl ether. Yield: 75.9% (1.58 g). ^1^H NMR (CDCl_3_): 7.1 (d); 7.3 (t); 7.4 (m); 7.5 (t); 7.7 (d); 7.8 (d); 8.1 (d); 9.4 (d). IR (KBr, cm^−1^): 1621 (s), 1565 (s), 1469 (s), 1417 (w), 13218 (s), 1243 (w), 1173 (s), 969 (w), 878 (w), 821 (s), 734 (s). ESI-MS: (C_28_H_20_N_2_O_2_) MW = 416.47 g/mol. *Ms*/*z* (M + 1H^+^) = 417.16.

### 2.2. [(C_28_H_18_N_2_O_2_)_3_Dy_2_H_2_O] *(**1**)*

A stirred solution of SYMLH_2_ (0.084 g, 0.2 mmol), $\mathrm{Dy}{\left({\mathrm{NO}}_{3}\right)}_{3}{\mathrm{xH}}_{2}O$ (0.070 g, 0.2 mmol) and ${\mathrm{Et}}_{3}N$ (100 µL, 0.6 mmol) in $CH_{3}CN$ (20 mL) was heated under reflux for 2 h. After 5–6 days, orange crystals of **1** were obtained by slow evaporation of solution. Yield: 5.68% (6 mg). MALDI-TOF-MS: $\left[{\left(C_{28}H_{18}N_{2}O_{2}\right)}_{3}{\mathrm{Dy}}_{2}H_{2}O\right]$ MW = 1585 g/mol; *Ms*/*z* (M + 1H^+^ − $H_{2}O$) = 1568.

### 2.3. Iron Oxide Nanoparticles (IO-NPs)

The synthesis of iron oxide nanoparticles was carried out by modification of a published procedure [12]. Iron oleate (2.78 g, 3 mmol), oleic acid (0.96 mL, 3 mmol) and eicosane (10 mL) were mixed in a three-neck round bottom reaction flask and heated to 60 °C to melt the solvent. Then, the reaction mixture was heated to 336 °C at a rate of 3.3 °C/min, under stirring and with continuous refluxing for 10 min. Finally, the mixture was cooled down to 50 °C. To precipitate the IO-NPs, a mixture of 40 mL of acetone and 10 mL of hexane was added to the reaction flask. The IO-NPs were separated by centrifugation and washed three times. For long-term storage, the centrifuged IO-NPs were submerged in chloroform.

### 2.4. Decoration of Iron Oxide Nanoparticles (NP-1)

Fifteen milligrams of precipitate IO-NPs were shaken with 3 mg of complex **1** in chloroform for 72 h. The decorated nanoparticles were magnetically separated and washed with chloroform three times, to eliminate the remaining complex.

### 2.5. Characterization

Infrared spectra were collected using a KBr pellet on a Thermo-Nicolet AVATAR 330 FT-IRHih, at the Department of Inorganic and Organic Chemistry, Inorganic Chemistry section, University of Barcelona. Single crystal X-Ray diffraction was recorded on a Bruker (Karslruhe, Germany) APEXII SMART diffractometer, using Molybdenum Kα microfocus (λ = 0.71073) as a radiation source. The structure of complex **1** was resolved by intrinsic phasing (SHELXT) and refined in F2 (SHELX-2014). Crystallographic data can be obtained free of charge from the Cambridge Crystallographic Data Centre (CCDC deposition number 1842829, https://www.ccdc.cam.ac.uk/structures/).

Magnetic measurements were performed at the Unitat de Mesures Magnètiques of the University of Barcelona on a Quantum Design MPMS XL (USA) superconducting quantum interference device (SQUID) magnetometer equipped with a 5 T magnet. Diamagnetic corrections for the sample holder and for the sample using Pascal’s constants were applied. Hysteresis loops were measured using the hysteresis mode on a Quantum Design MPMS XL superconducting quantum interference device (SQUID) magnetometer equipped with a 5 T magnet. SQUID precision in a magnetic measurement requires a stable magnetic field; the delay for field stabilization is 5 s. The average field-sweep rate is 3 mT/s.

IO-NPs were characterized at the Serveis Cientifico-tècnics of the University of Barcelona using a JEOL J2100 (LaB6 filament) Transmission Electron Microscope (USA) operating at 200 kV fitted with GATAN digital camera (Gatan Inc., Pleasaton, CA, USA). Gatan Digital Micrograph software was used to process and analyze high-resolution TEM (HRTEM) images.

### 2.6. Computational Methods

All electronic structure calculations were carried out with Gaussian09 [17] revision D.01 at the DFT level of theory. The molecules of hexane, benzene, propene and naphthalene were fully optimized using the B3LYP [18] functional with the 6-31G(d) basis set [19] for all atoms. Optimizations of the adducts, composed of two monomers, were performed at the ωB97Xd [20]/6-31G(d,p) level to capture the dispersive nature of the interaction. On the other hand, the geometry of the oleic acid model was optimized using a Grimme dispersion [21]-corrected DFT method denoted as B3LYP-D3/6-31G(d,p). This dispersion correction energy term is a relatively simply function of interatomic distances that contains adjustable parameters fitted to many computed interaction energies. Dispersion corrections can lead to significant improvements in accuracy with a negligible computational cost. Since the oleic acid model pretends to mimic the geometry adopted by the organic chains anchored onto the magnetic NPs surface, during the optimization process, the carboxylic oxygen atoms were kept frozen.

All calculated monomers and adducts were characterized as real minima of the potential energy surface (PES) by vibrational analysis. The interaction energies corresponding to all adducts were calculated as the difference between the total electronic energy of two monomers and the adduct, $\Delta E=\left(E_{\mathrm{monomer},\;1}+E_{\mathrm{monomer},\;2}\;\right)-E_{\mathrm{adduct},\;1-2}$. The interaction energies were corrected for the basis sets superposition error (BSSE) by the Counterpoise method [22]. The tetrameric organic model was not characterized as real minima of the PES because our final goal was to analyse how the oleic acid chains are reorganized when immobilized on the iron oxide surface of the nanoparticle. 

## 3. Results and Discussion

### 3.1. Synthesis and Characterization

**Scheme 1:**
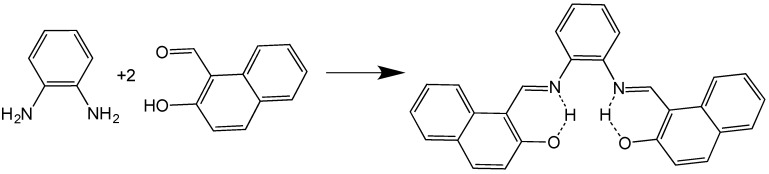
Synthesis of the Schiff-base ligand SYMLH_2_.

The Schiff-base ligand (SYMLH_2_) had been previously synthesized and characterized by Sañudo and Gholizadeh [6], following a reported procedure [23]. The ligand $C_{28}H_{20}N_{2}O_{2}$ (SYMLH_2_) was synthesized by the condensation reaction of a *N*,*N*′-bis(1-naphthaldiamine)-*o*-phenylenediamine with two equivalents of 2-hydroxy-1-naphthaldehyde in ethanol under reflux (Scheme 1).

A good yield of SYMLH_2_ was obtained and was used without further purification. The ligand purity was checked by ^1^H-NMR, IR, and mass spectroscopy. It is a symmetric organic molecule that presents one coordination pocket that should lead to sandwich-type complexes with lanthanide ions. The presence of aromatic rings provides a highly-concentrated electronic density throughout the ligand plane, which also makes it ideal for forming sandwich-type metal complexes [5]. The quasi-planar geometry of the ligand can lead to a proper deposition over a surface. Due to the relatively simple yet robust synthetic procedure, a wide variety of Schiff base ligands can be designed with specific chemical groups to anchor them to surfaces, and for generating tailor-made coordination complexes.

The reaction of the SYMLH_2_ with Ln(NO_3_)_3_ (Ln = Er, Yb) was performed previously in the group using acetonitrile as a solvent and excess of trimethylamine to deprotonate the two groups of the ligand [6]. A priori, we expect that Dysprosium (III) nitrate hydrate to react in a similar way with the ligand. Similar complexes have also been reported by Gorden et al. [24]. An MeCN reaction mixture was prepared with a stoichiometric ratio 1SYMLH_2_:1Ln. Orange crystals of SYML-Dy2 were isolated by slow evaporation after a few days and their crystal structure was confirmed.

**Figure 1:**
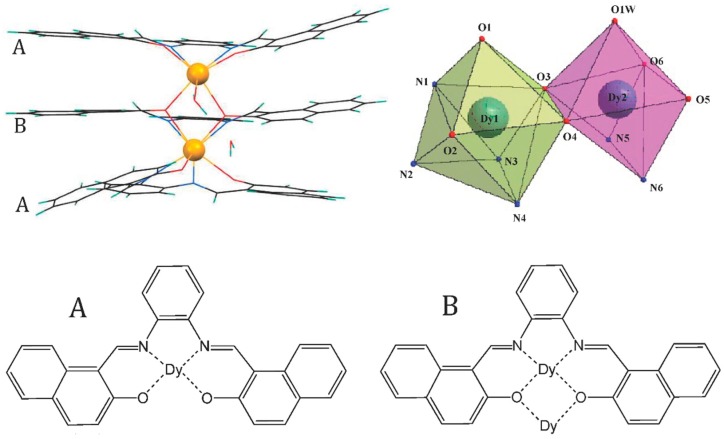
Crystal structure, polyhedral around Dy of SYML-Dy2 and ligand coordination modes **A** and **B**, Dy: yellow, O: red, N: blue, C: black and H: green.

Single-crystal X-Ray diffraction analysis reveals that the SYML-Dy2 crystallizes in a triclinic system with the space group P-1. Crystallographic data and data collection details for the SYML-Dy2 complex is presented in Table 1. The complex can be described as a double-double decker complex, or double sandwich, where the Schiff base ligands actively use the two oxygen atoms to bridge two Dysprosium (III) ions (Figure 1).

The coordination spheres of the two Dy^+3^ in the complex are not equal. As shown in Figure 1, one lanthanide ion, Dy1, is located in an O_4_N_4_ coordination pocket of two deprotonated SYMLH_2_ ligands. While the other lanthanide ion, Dy2, stays in a O_5_N_2_ coordination sphere with two nitrogens and four oxygen atoms of the SYML ligands, and one coordinated water molecule. The Dy1 atom is octacoordinated with a distorted square antiprism (SAP) coordination polyhedron, whereas the Dy2 is heptacoordinated with a distorted capped trigonal prism coordination polyhedron. The SYML-Dy2 complexes are stacked along the a-axis of the unit cell, displaying π–π stacking interactions between the ligands’ naphtalene groups. There is a water of crystallization, O2w, which is hydrogen bonding to the terminal water O1W, with O–H···O distance of 2.698 Å. These are the most relevant intermolecular interactions in the crystal structure of SYML-Dy2. O1w and O2w establish much weaker interactions with phenoxo O from an SYML ligand in coordination mode A of two neighbouring complexes. The O–O distances are d(O1w-O5) = 2.839 Å, d(O2w-O1) = 2.937 Å and d(O2w-O6) = 2.877 Å.

This sandwich triple decker type structure resembles the dinuclear lanthanide phthalocyanine sandwich complexes [25] of the first reported lanthanides mononuclear SMMs. The first studies based on these f-electronic systems showed that the suitable choice of a ligand field can lead to a highly anisotropic ground state [5].

### 3.2. Magnetic Properties

**Figure 2:**
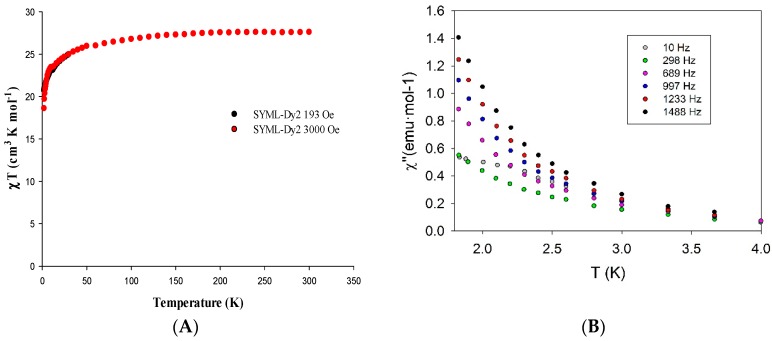
(**A**) Magnetic susceptibility plot for SYML-Dy2. (**B**) Out-of-phase alternate current (AC) magnetic susceptibility for SYML-Dy2, with an applied direct current (DC) field of 2000 Oe.

Magnetic susceptibility data were collected for the SYML-Dy2 complex at two different applied dc fields, 193 Oe and 3000 Oe, in the 2–300 K temperature range. As shown in Figure 2, the experimental χT value of 27.56 cm^3^ K mol^−1^ at 300K is in good agreement with the expected values for the proposed complex formula. Since the expected value for uncoupled Dy (III) ions (^6^H_15/2_, S = 5/2, L = 5, J = 15/2 and g_J_ = 4/3) [26] is 14 cm^3^ K mol^−1^, the experimental value for the SYML-Dy2 complex with two Dysprosium atoms is almost double. The χT product is non-field dependent, because data measured at two different dc fields overlap. As the temperature goes down, the χT product slowly decreases and shows a sharper decrease below 50 K. The changes in χT product with temperature are to be expected, due to the Boltzmann depopulation of M_J_ sublevels and the weak magnetic coupling which is typical for Dy complexes [4]. Magnetization vs. field data at 2 K show a maximum value at 5 T of M = 11.15 $\mu_{B}$, and the lack of saturation on the M vs. H data at 2 K suggests the presence of a significant anisotropy and/or low-lying excited states [27].

The dynamics of relaxation of magnetization were investigated using alternate current (AC) susceptibility measurements. In order to observe the ac out-of-phase signal, dc fields had to be applied. The best field was 2000 Oe, since at higher fields, the maximum was not observed and the signal was weaker. Figure 2 depicts out-of-phase (χ″) signals vs. T under a dc field of 2000 Oe. The data display frequency dependence in the out-of-phase signal. This behavior indicates slow magnetization relaxation, so the complex can be interesting as new field induced SMM. However, a maximum of χ″ was not observed, so the characteristic relation time of the system cannot be calculated. The complex SYML-Dy2 does not show hysteresis of the magnetization at temperatures above 1.8 K. A Cole-Cole plot can be used to extract relaxation times. However, for SYML-Dy2 the Cole-Cole or Argand plot clearly shows the onset of a slow relaxation process at very low frequencies (below 100 Hz) that overlaps with the fast relaxation process, precluding the treatment with a Debye model to extract relaxation times. The data are shown in the Supplementary Information.

### 3.3. Nanostructuration of SYML-Dy2 on Iron Oxide Nanoparticles

A challenge in molecular electronics is to control the magnetic coupling between magnetic molecules and magnetic electrodes, in order to build efficient devices [28]. In this research, we use well-known iron oxide nanoparticles as the support for a hybrid material containing a 4f molecular nanomagnet. The system is easy to manipulate in solution and it is a good model for a magnetic surface. Furthermore, the IO-NPs used in this study are decorated with oleic acid, the role of which is to address the decoupling of the SMM to the surface. The synthesis of the NPs in surfactant-containing solutions like oleic acid or dopamine provides proper nanoparticle stabilization, prevents particle aggregation, and avoids surface oxidation of magnetite [29].

**Figure 3:**
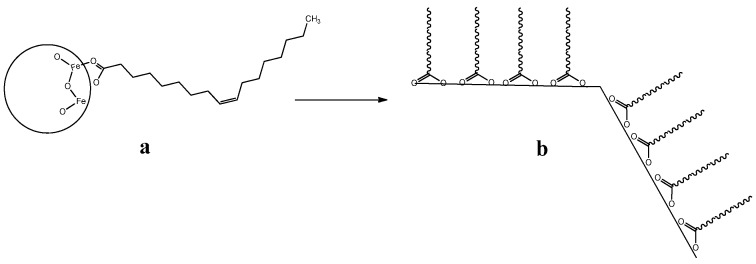
Scheme of the hybrid system. (**a**) Carboxylic groups are bonded unequally. (**b**) Two kinds of oleate chains: those on the facet of the iron oxide crystal and those on the edge.

The nanoparticles were prepared using a well-established procedure in the literature by high-temperature decomposition of the iron oleate complex with oleic acid as a surfactant [12]. This procedure has been widely used and yields crystalline superparamagnetic nanoparticles of magnetite, the surfaces of which are capped with an oleic acid monolayer. In order to elucidate how the carboxylic group of the oleic acid interacts with the superficial iron atoms, the NPs were analyzed by infrared spectroscopy (IR). Clear peaks expected for *syn*,*syn*-carboxylato groups are found, indicating that both oxygens of oleate are coordinated to the Fe ions of the NP surface (Figure 3).

**Figure 4:**
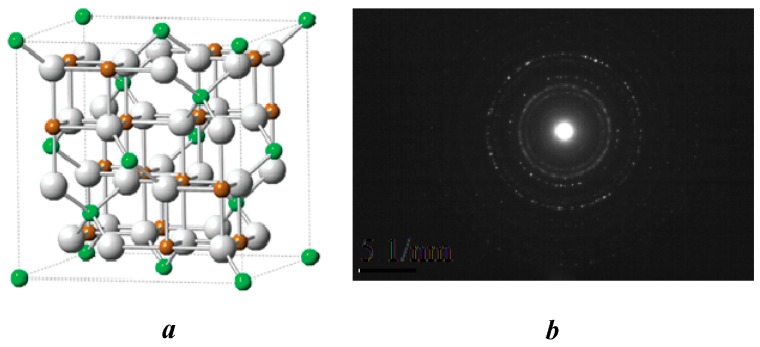
(**a**) Crystal structure of magnetite, Fe: green, orange; O: grey. (**b**) Diffraction pattern of the iron oxide nanoparticles (IO-NP).

Magnetite is an ideal oxide support; it is easy to prepare, with a very active surface that has a cubic inverse spinel structure (Figure 4). The unit cell has interesting properties, because the presence of non-equivalent cations in two valence states, Fe^+2^ and Fe^+3^, in the crystal structure leads to the formation of a unique magnetic structure. The unit cell also contains 32 O^−2^ ions which are regular cubic close packed along the [110] direction. Generally, Fe_3_O_4_ crystals are distributed with octahedral and mixed octahedral/tetrahedral layers along the [111] direction. As shown in Table 2, the distances of the XRD (X-Ray diffraction) pattern (Figure 4) of the synthetized NPs fit with magnetite simulations.

**Figure 5:**
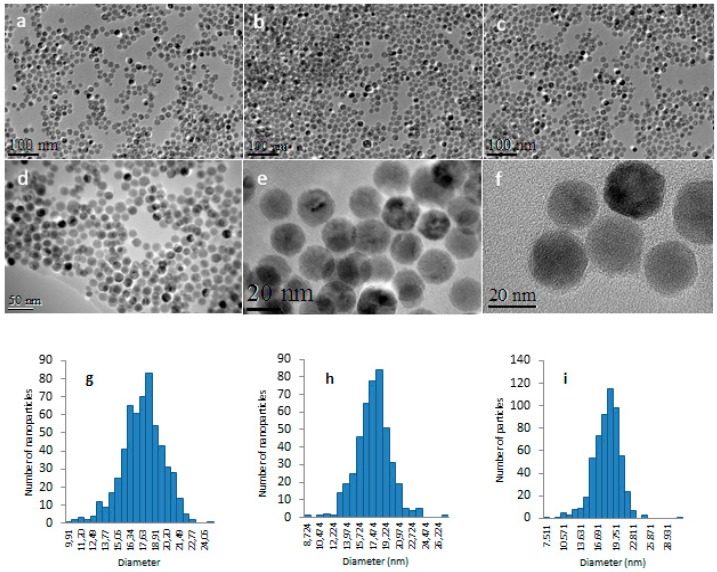
(**a**–**f**) Transmission electron microscope (TEM) images of IO-NPs. (**g**–**i**) Histograms of the a, b and c TEM images respectively. The mean diameter and standard deviations are: (**g**) 17.264 nm ± 2.3%, (**h**) 17.087 nm ± 2.2% and (**i**) 17.339 nm ± 3.28%.

One of the main advantages of this synthetic procedure is that iron oxide NPs can be prepared in a wide range of sizes (from 6 to 30 nm) by simply varying the reaction conditions. The transmission electron microscopy (TEM) technique allows us to test not only the size, but also the morphology and the polydispersity of the obtained NPs. The TEM images presented in Figure 5 show an average NP diameter of 17 nm, with narrow particle size distribution, which means the NP sample is monodisperse. As noted in the TEM images, the synthetized IO-NPs are not exactly spherical; they have a polyhedral shape. The existence of these well-defined faces allows us to distinguish between two kinds of oleic acid chains: those that are located in the middle of the faces and those located on the edges of the NPs (Figure 5b). The latter are easy to remove, because they do not interact with adjacent chains in all directions. The oleic acid that are in the middle of each face of the NP are surrounded in all directions with other oleic acids in a SAM; thus, the attractive CH···HC interactions (see the theoretical calculation section) that stabilize the SAM are maximized for these, but not for the oleic acid molecules on the edges. Thermogravimetric analysis shows these relative energies, since two clearly different losses of organic matter at two temperatures take place. At 516 °C the first drop in the curve occurs, corresponding to a loss of 3.65% of organic matter; the second occurs at 768 °C, corresponding to a loss of 4.68%.

### 3.4. Decoration of Nanoparticles

To obtain the hybrid system, the oleic acid NPs are functionalized with the SYML-Dy2 complex, following a standard method used in the group [29]. SYML-Dy2 was added to an NP chloroform solution and shaken for three days, in order to allow grafting to occur within the complex. The resulting sample was separated by decantation using a hard magnet, and the solid was washed several times to ensure that no traces of unattached SYML-Dy2 remained.

**Figure 6:**
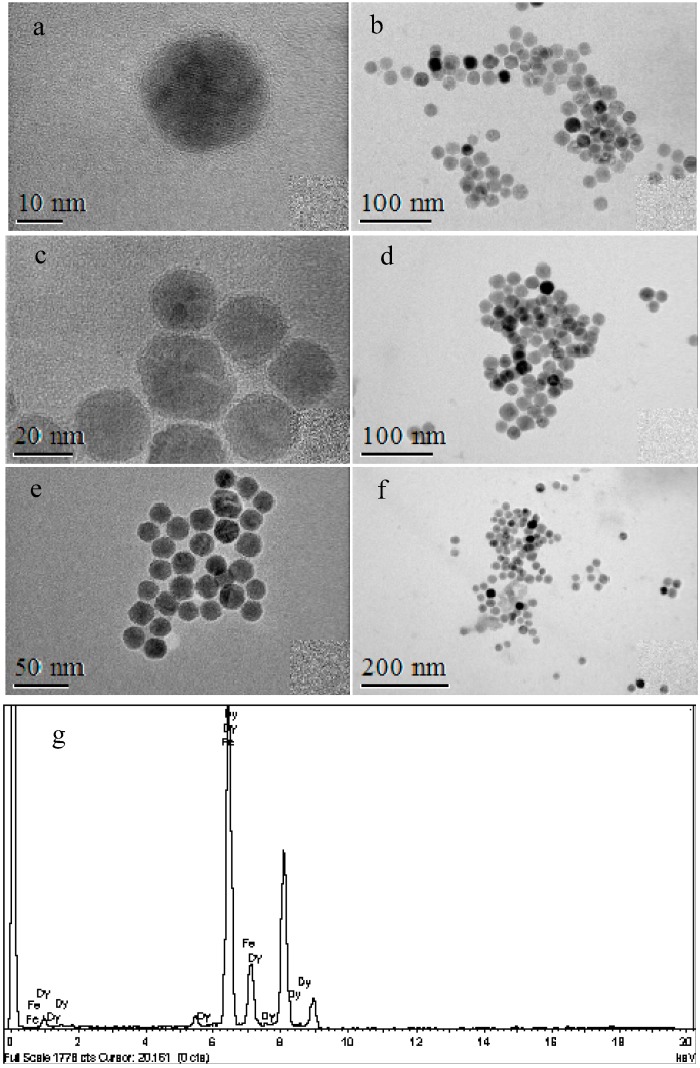
(**a**–**f**) TEM images of IO-NPs decorated with SYML-Dy2. (**g**) Energy-dispersive X-Ray spectra of NP with SYML-Dy2.

The TEM images (Figure 6) of the IO-NPs loaded with SYML-Dy2 showed that the size distribution was similar to that of the unloaded NP sample. Decorated NPs also maintained their shape and crystalline structure, which demonstrates that the decoration process does not affect the NP sample. Energy dispersive X-ray analysis (EDX) performed on the decorated NP-Dy2 showed a low signal of Dy (Figure 6g), which can be explained due to the small percentage for Dy2-SYML with respect to the amount of iron oxide in the NP. 

### 3.5. Magnetic Properties

**Figure 7:**
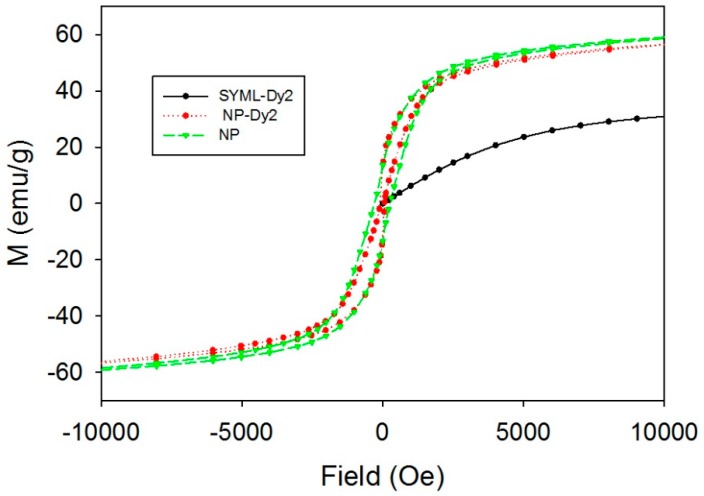
Hysteresis of the magnetization vs the applied field for NP, NP-Dy2 and magnetization vs. field for the complex SYML-Dy2 shown in emu/g at 2 K.

Magnetization vs. field data at 2 K were collected for the oleic acid NP and the hybrid NP-Dy2, to determine whether there were interactions between the SMMs and the decorated IO-NP. The data are presented in Figure 7, as magnetization per gram of material vs. field. Clearly, the NP magnetism dominates the bulk data. In the hysteresis cycle at 2K (Figure 7), the NP-Dy2 system presents a saturation magnetization of 67.72 emu/g material, while the maximum value of magnetization for oleate IO-NP is 69.88 emu/g material. Taking into account the fact that our studied system contains some percentage of organic matter, a total magnetization value can be calculated. The amount of organic matter in both samples can be deduced from thermogravimmetric analysis (TGA) experiments. From TGA, the total amount of organic matter in both samples is reflected by a total loss of 8.33% and 12.11% of IO-NPs and NP-Dy2 respectively. The saturation magnetization values per gram of oxide are 76.23 emu/g oxide and 77.05 emu/g oxide for IO-NP and for NP-Dy2 oleic acid NPs, respectively. If we take into account the M_s_ per gram of iron oxide, the hybrid system value is higher than that of the IO-NP. This is due to the fact there are SYML-Dy2 molecules in our sample that have a huge value of magnetization per gram, and thus, the SYML-Dy2 magnetization must be taken into account if functionalization with SYML-Dy2 has been successful. The obtained values for the saturation magnetization of the NPs are similar to that of the bulk magnetite; this value can range from 80 to 100 emu/g oxide [30]. Nevertheless, as expected, the NPs still have lower M_s_ than bulk iron oxide, due to the surface effects. An oxidation process to maghemite at surface level is expected for NPs of this size [31].

Hysteresis loops were measured on a commercial SQUID using the hysteresis mode. In Figure 7 the hysteresis loop of each sample can be observed. In fact, in the NP-Dy2, a step can be observed that gives rise to a kind of butterfly loop, which is attributed to QTM [32], due to the level degeneracy between M_J_ states. The coercive field of the hysteresis loop at 2K for the IO-NP was 560.86 Oe, while the hybrid NP-Dy2 had a coercive field of 177.2 Oe, due to the QTM step at zero field. QTM is very frequent in lanthanide SMMs [4], so we can confirm that the tunneling effect of the Dy (III) SMMs is present in the hybrid NP-Dy2 system. The interaction between the NP and the SYML-Dy2 SMM shell should be dipolar in origin, accepting that the SYML-Dy2 complex is sitting on the oleic acid layer. This is supported by a favorable interaction, as shown by the model calculation and the TGA results [33,34].

Even though the calculations suggest that there should be a unique orientation of the SYML-Dy2 molecule to maximize the favorable interactions with the aromatic rings of the ligand, one must consider the possibility of a distribution of distances between NP and SYML-Dy2 due to different dispositions of the SMM on the NP surface, i.e., a distribution of fields felt by the molecules SYML-Dy2. The typical dipolar field for a 5 nm iron oxide NP was determined by Moya and co-workers to be 114 Oe at a distance of 1 nm from the iron oxide [35]. Similar values can be expected in the present system.

Even such a weak interaction can still affect the hysteresis loops of the hybrid system, as observed here. The main effects are lower saturation magnetization per gram of sample and the QTM step at zero field in the hysteresis loop, typical of lanthanide SMMs. In our previous paper, we reported Ni_4_Tb SMMs grafted onto Fe_3_O_4_ NP [25] via a dopamine linker. We observed changes in the hysteresis loops of the hybrid material caused by the effect of the molecules via the organic layer of dopamine. An enhancement of the magnetic properties of maghemite (Fe_3_O_2_) NP has been observed by Prado et al. by coordination of a Co(II) coordination complex to Fe_2_O_3_ NPs [15]. They propose that covalent linking of the two species through oxo-bridges, and the resulting magnetic interaction, are key for the observed enhancement of the magnetic properties. In our hybrid system, there is no direct covalent bond between the SMM and Fe, and the dipolar magnetic interaction between Dy(III) in SYML-Dy_2_ and the Fe_3_O_4_ results in a significant enhancement of QTM.

### 3.6. Theoretical Study

Next, we carried out a theoretical study to try to understand the features of the hybrid system composed of the magnetite NP, the capping oleic acid monolayer, and the Dysprosium triple-decker complexes atop such a monolayer. We narrowed our focus to the intermolecular interactions that dictate the conformation of the capping oleic acid monolayer, and also in the interface between such a monolayer and the triple-decker complex supported on it. We have designed two simplified models to analyse the lateral interaction between the fatty acids chains on one side, and between the terminal part of the fatty acid and the aromatic part of the Dy compounds on the other. The main goal of this computational analysis is to determine the topology of the oleic acid monolayer and the means by which the metallic complexes can be supported on it, keeping in mind that this is a very simplified model that does not aim to yield an in-depth understanding of the NP-oleic acid hybrid system.

**Figure 8:**
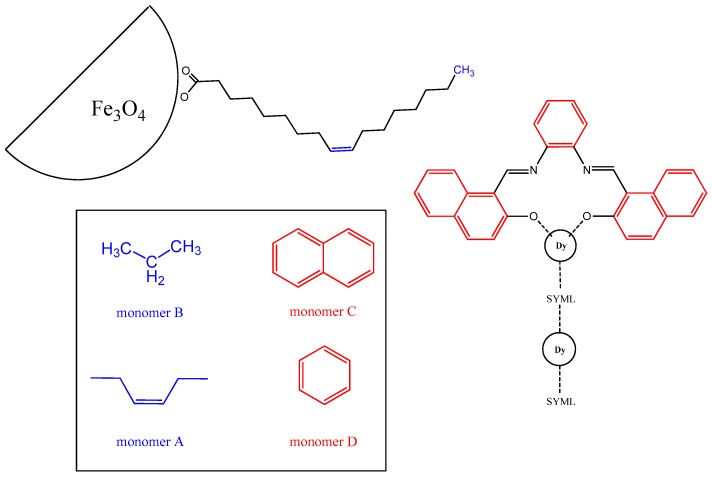
Schematic representation of the oleic acid monolayer-Dy complex interface, with the corresponding units that can interact with each other.

As mentioned above, magnetite NPs are often bound to molecule layers to generate nano-conjugates that can be used as versatile hybrid systems. In our system, the magnetite surface is capped by a monolayer of oleic acid. As revealed by the IR spectra, one of the oxygen atoms at the carboxylic group of the acid presents a stronger interaction with the NP surface, specifically, with the most accessible Fe^2+^ cation. In our first model, we consider that the fatty acid molecules are attached to the surface at the positions of the Fe^2+^ cations on the [001] face of the Fe_3_O_4_, i.e., separated by 8.43 Å, assuming a lattice constant a = 0.839 and that the NP surface is completely planar, at least on each crystal facet (Figure 8). The fatty acid in our model is (*E*)-dec-5-enoic (C_10_H_18_O_2_), to reduce the computational cost. A tetramer, consisting of four fatty acids forming a rhomboid, was optimized, while the coordinates of the O atoms involved in the Fe–O bonds were kept frozen to simulate the presence of the magnetite surface. The main interactions between the acid molecules are homopolar dihydrogen contacts of the type C–H···H–C. After optimization, the double bonds are oriented in the same direction with respect to the face, and therefore, the terminal methyl groups are all located at practically the same distance (≈11 Å) from the NP surface. The H···H intermolecular distances are between 2.3 and 2.5 Å, in agreement with previously calculated attractive dihydrogen interactions [36,37,38,39,40,41]. It has been also seen in previous studies that multiple H···H interactions working together can lead to surprisingly strong interactions [42].

The fact that all fatty acids show the same height once deposited on the NP creates a pseudo-surface of methyl groups on the exposed part of the molecules. Such an extended surface of terminal alkanes should be, a priori, able to interact with the aromatic part of the triple-decker Dysprosium complex. On the other hand, in the NP edges the double bond of the oleic acid, molecules should be exposed (within a facet it is protected by the alkyl groups), and thus, also able to interact with the metallic complex. Therefore, the theoretical characterization of this interface (Monolayer-Dy complex) must include the interaction between the aromatic complex ligand and the alkane and alkene parts of the oleic acid. In this context, we have created a very simplified model that simulates all possible interactions between the potential reactive groups (Figure 8). The fatty acid may interact either by the *cis*-double bound, modeled as *cis*-3-hexene (monomer A), or through the terminal methyl group, modeled as a propane molecule (monomer B), while the SYML ligand presents the naphthalene (monomer C) and benzene (monomer D) groups as the potentially interacting ones.

The geometry of the four possible adducts formed by the monomers has been optimized at the ωB97xD/6-31G(d,p) level. The calculated interaction energy values lie within the range of weak noncovalent interactions, which typically range from a hydrogen bond in water (aprox. −5 kcal/mol) to a very weak induced dipole-dipole interaction of −1 kcal/mol. Adducts A–C and B–C are the most stable ones, having the expected energy values based upon interaction type. However, the interaction in adducts A–D and B–D is still attractive, the interaction energy being −3.60 and −2.63 kcal/mol, respectively. The adduct A–C presents π−π staking interactions of −5.67 kcal/mol, while the adduct B–C forms a C–H⋯π interaction of −4.98 kcal/mol. Therefore, we can conclude that the complex interacts with the organic chain through the naphthalene/benzene groups of the ligand. Since the adduct A–C present the most favourable interaction energy, we would expect naphthalene to be deposited on the monolayer by interacting with the *cis* double bound of fatty acid. However, that interaction is not sterically favoured, because the double bond is not exposed to the surface. As the NPs have a polyhedral shape, we expect this more stable interaction in the edges of the faces, where the *cis* bound is not protected, while the interaction between the aromatic rings of the ligand and the methyl group (adduct B–C) takes place on the faces of the NPs. In summary, thanks to our computational analysis, we can conclude that the interaction between the oleic acid monolayer and the Dysprosium complexes is favourable in terms of energy, which should facilitate the spontaneous deposition of the latter on the former. Many papers in the literature deal with the well-known TbPc SMM on surfaces. It is relevant to mention here that the molecules may suffer strain or deformations while on the surface, or that several orientations of the molecule on the surface are possible. This is particularly so when STM is used to characterize the nano-objects, or the molecule is constrained between break junction electrodes [43,44,45].

## 4. Conclusions

The SYML-Dy2 was grafted intact onto a magnetic surface and maintained its properties, giving rise to a nanostructured hybrid system composed of iron oxide nanoparticles. One of the key points of this nanostructure is the oleic acid monolayer around the NP, which decouples the SMM of the surface. SQUID data revealed the tunneling effect of the Dy (III) SMM in the hybrid system. This opens up great possibilities in nanostructured materials of molecular systems, since it should now be possible to effectively control coupling, or to decouple the SMMs from the substrate, and thus, observe the inherent bistability of SMMs on a surface.

The nanostructured system has been also characterized theoretically using computational tools. The geometry adopted by the organic chains was optimized at the DFT level, and the existence of noncovalent interactions between the molecule and the NP was proven. The capability of the alkyl chains to establish lateral dihydrogen interactions dictates the conformation of the oleic acid monolayer attached to the NP. Moreover, this methyl-terminated monolayer is able to engage in attractive interactions with the planar aromatic region of the Dy complex, highlighting the role of noncovalent interactions in the stability of nanostructured hybrid systems.

## Figures and Tables

**Table 1:** Crystal data collection and structural parameters for Syml-Dy2 Complex.

Compound	SYML-Dy2
Crystal system	Triclinic
Space group	P-1
a/Å	10.5560(9)
b/Å	18.0269(14)
c/Å	18.4384(15)
alfa/°	66.851(4)
beta/°	73.702(4)
gamma/°	86.391(4)
Volume/Å^−3^	3092.1(5)
Z	2
Final R	$R_{1}\;$ = 0.0271 wR_2_ = 0.1048
T/K	100

**Table 2:** Comparison of experimental diffraction pattern data and simulation with magnetite.

Experimental (Å)	Simulation (Å)	Family of Planes
2.646	2.648	0 1 3
2.097	2.031	3 2 2
1.559	1.555	2 3 4
1.522	1.529	1 2 5

## Supplementary Materials

The following are available online.
