# Supplementary material for: Understanding the Molecule-Electrode Interface for Molecular Spintronic Devices: A Computational and Experimental Study
Source: Molecules. 2018 Jun 13;23(6):1441. doi: 10.3390/molecules23061441 (PMC6100063; doi:10.3390/molecules23061441)
Supplement: Supplementary file 1 [file molecules-23-01441-s001.zip › molecules-297482-SI.pdf]

Electronic Supplementary information for

Understanding the molecule-electrode interface for molecular spintronic devices: A computational and experimental study

Lidia Rosado Piquer,[a,b] Raquel Royo Sánchez,[a] E. Carolina Sañudo [a,b,]*

and Jorge Echeverría [a,c,]*

a-Departament de Química Inorgànica, Secció de Química Inorgànica, b- Institut de Nanociència i Nanotecnologia (IN2UB), c-Institut de Química Teòrica i Computacional (IQTC-UB) Universitat Barcelona, Martí i Franqués 1-11,08028, Barcelona.

SYMLH2 characterization: the synthesis of the ligand was performed following our reported procedure (New J. Chem., 2017, 41, 10101). NMR, IR and ESI-MS are in agreement with the data reported therein.

SYMLH$_2$ ^{1}H-NMR in $CDCl_3$

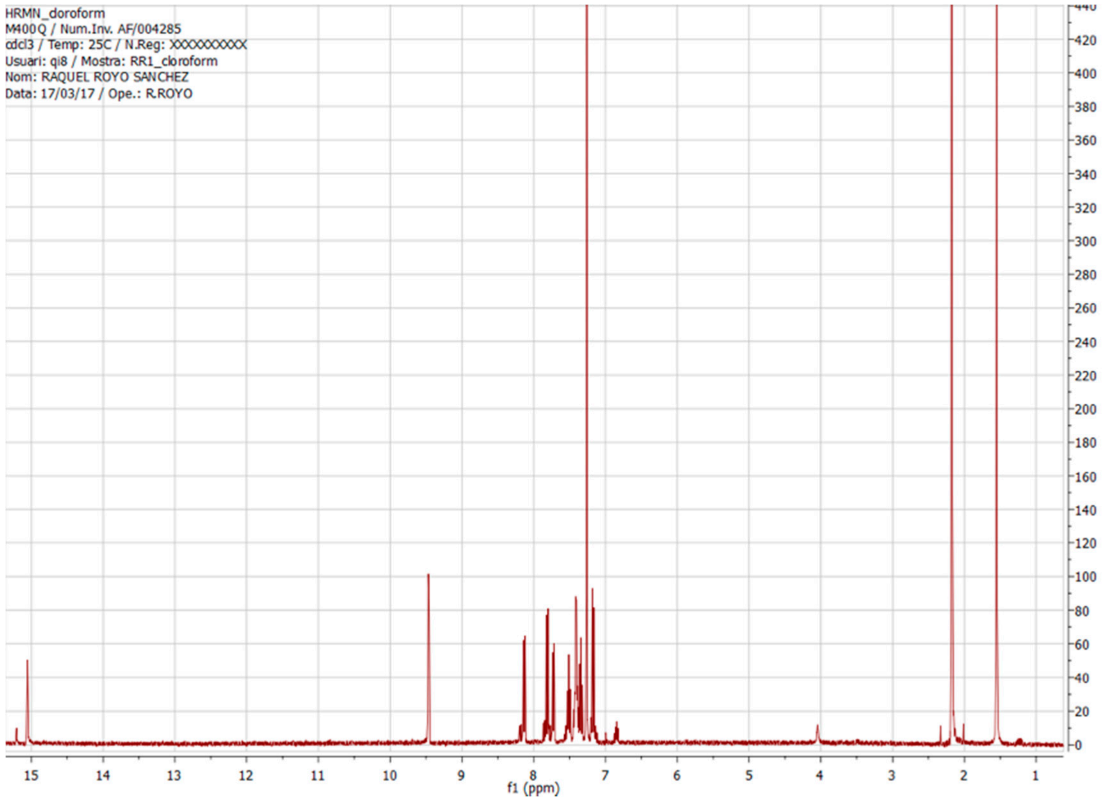

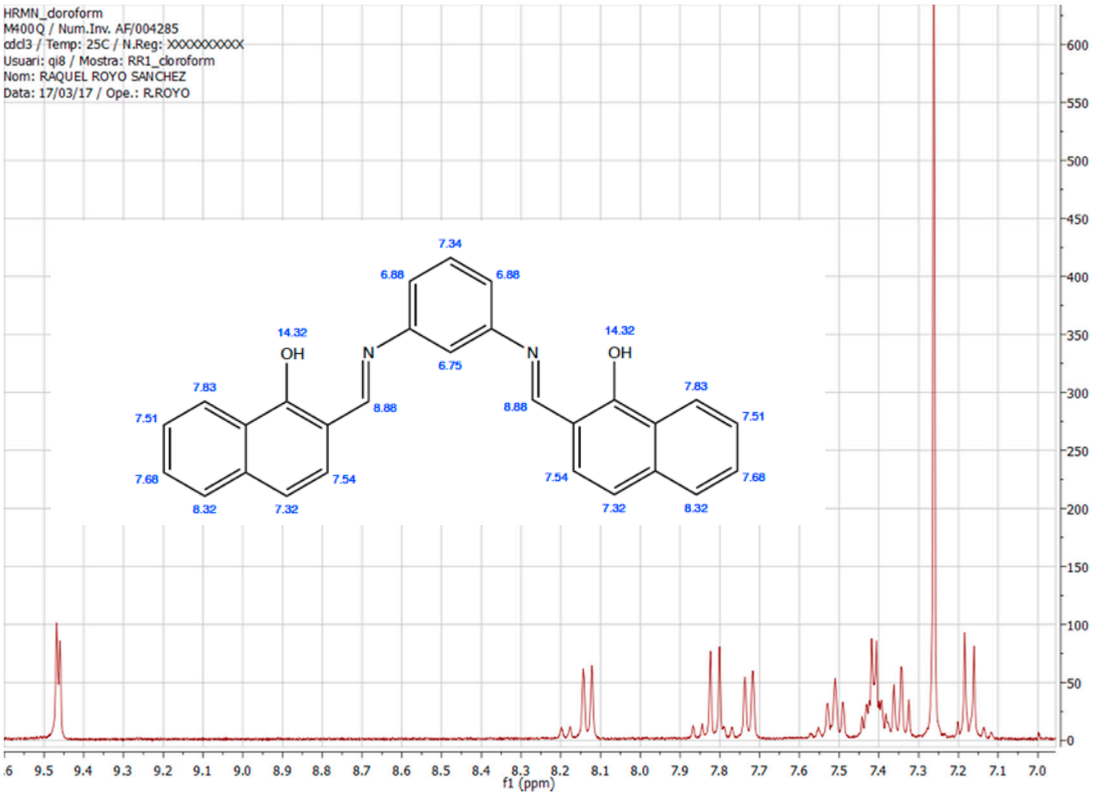

^{1}H NMR chemical shifts calculated using ChemBioDraw.

IR spectrum of SYMLH$_2$

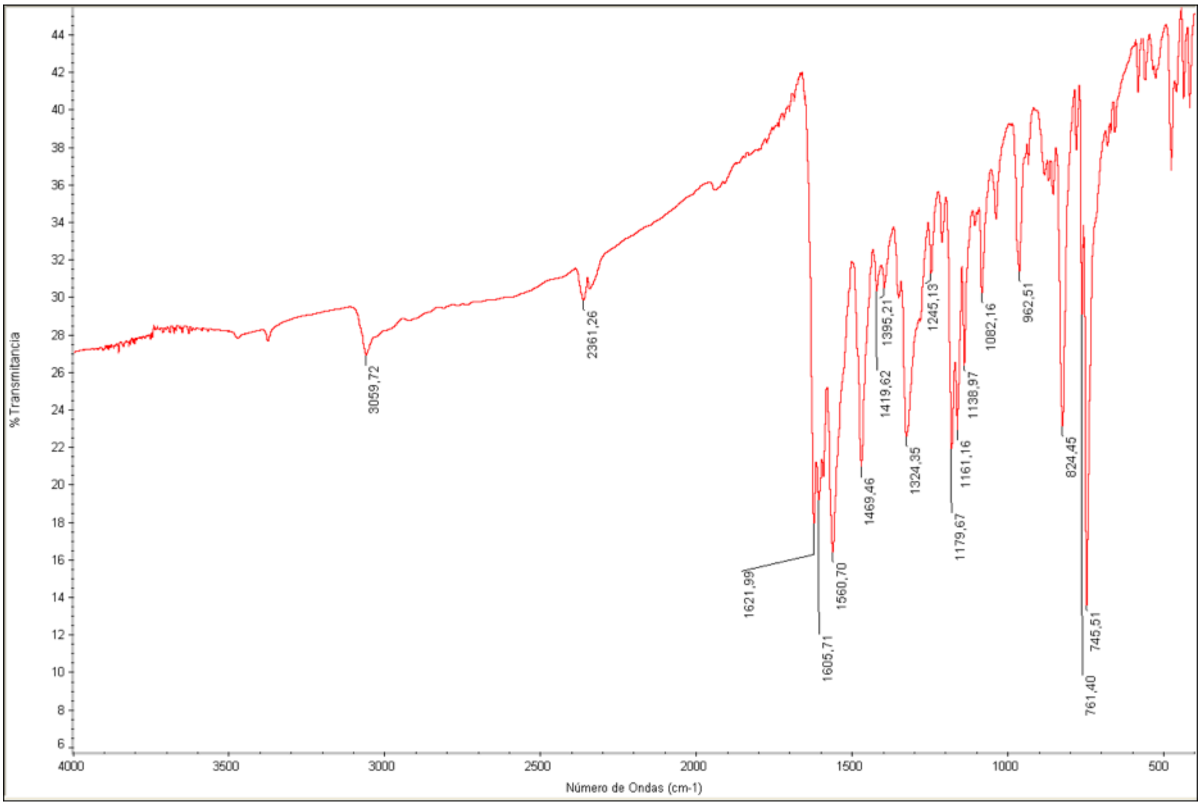

IR spectrum of iron oxide NP with oleic acid monolayer

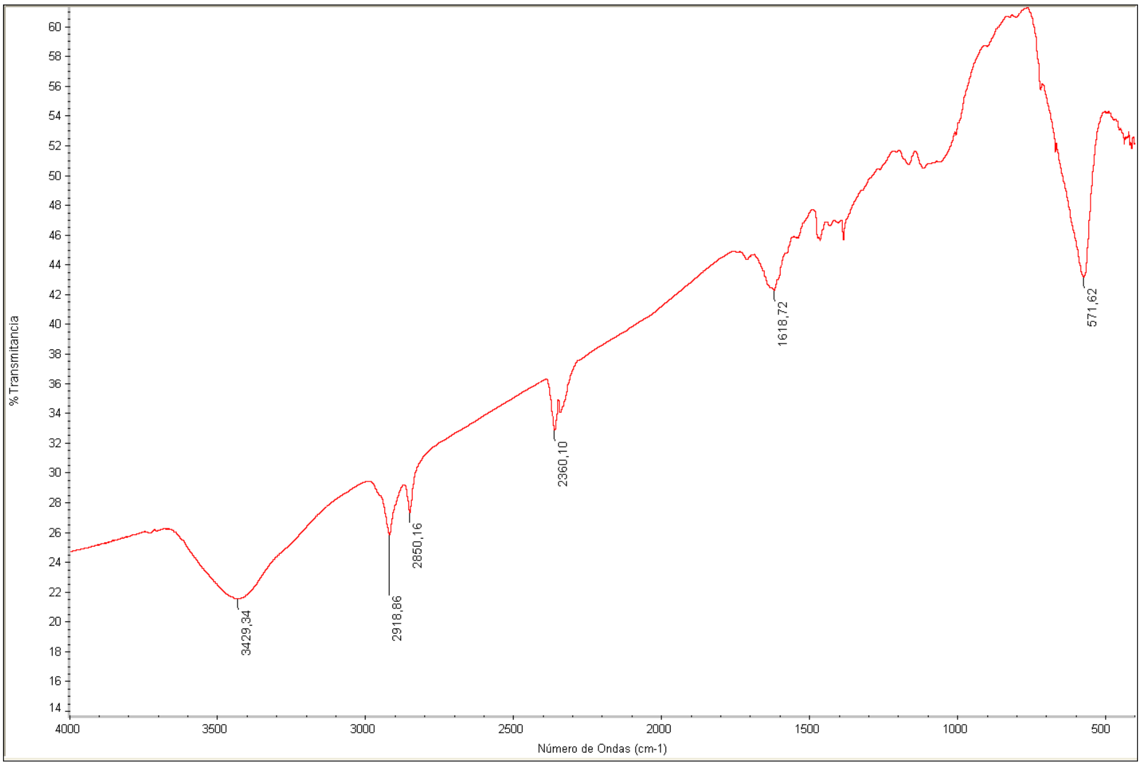

Magnetic properties of SYML-Dy2

Magnetization vs. field plot for SYML-Dy2 at 2 K.

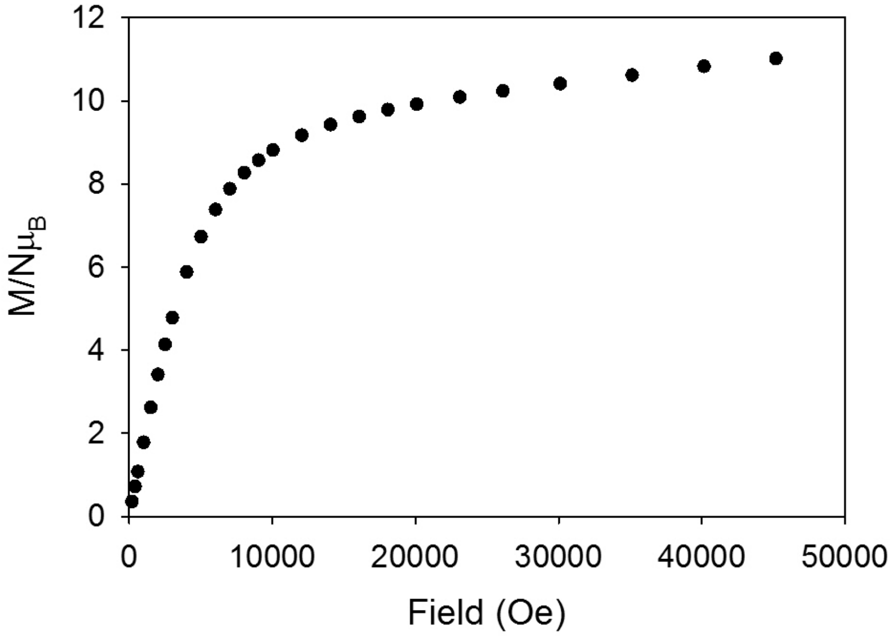

Out-of-phase ac magnetic susceptibility of SYMl_Dy2 at different applied DC fields at 1000 Hz.

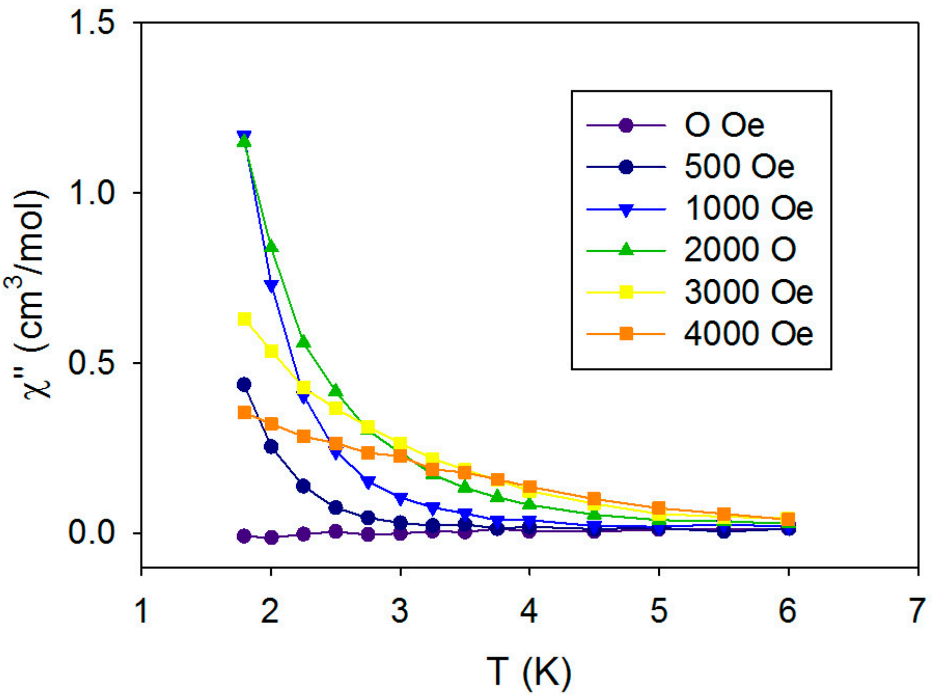

Out-of-phase AC magnetic susceptibility as a function of frequency with an applied DC field of 2000 Oe between 1.8 K (black) and 3.6 K (dark green).

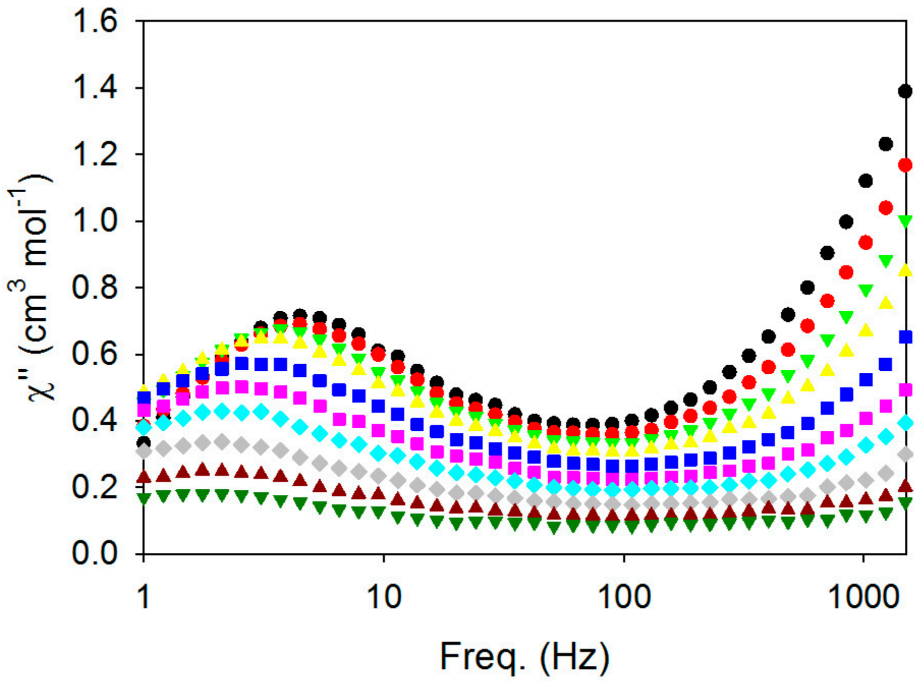

Argand plot for SYML-Dy2 with an applied dc field of 2000 Oe at the indicated temperatures with frequencies between 1 and 1500 Hz.

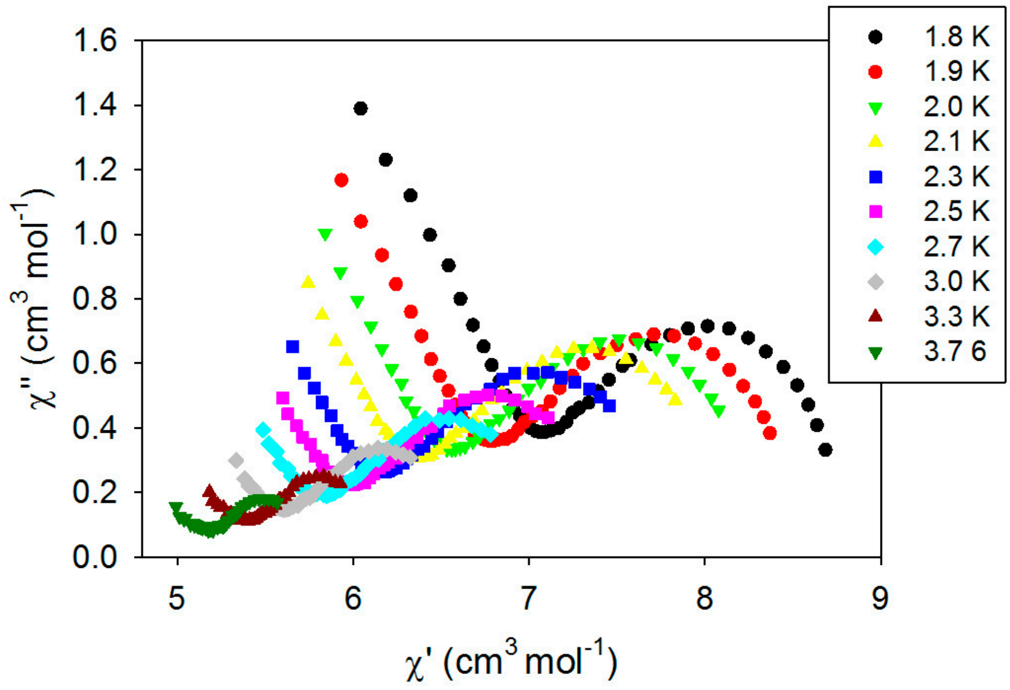

Cartesian coordinates of optimized systems.

Tetramer of (*E*)-dec-5-enoic acid

H	-1.001935	3.961386	-5.585817
H	2.176077	-1.003863	-4.704647
H	-2.866566	-4.148321	-4.236597
H	0.308807	-9.113530	-3.359415
O	-0.575805	6.114204	-4.591916
O	-1.065346	3.983892	-4.237494
O	2.602299	1.148749	-3.710355
O	2.112495	-0.981571	-3.356340
O	-2.440150	-1.995582	-3.242685
O	-2.929877	-4.125844	-2.888269
O	0.737891	-6.961665	-2.364983
O	0.246631	-9.091615	-2.011020
C	-0.908637	5.232868	-3.844264
C	2.441029	0.230742	-2.951561
C	-2.558920	-2.921188	-2.486537
C	0.482943	-7.856472	-1.605155
C	-1.232640	5.391452	-2.365228
H	-2.002847	6.171071	-2.321829
H	-1.670248	4.467763	-1.978011
C	-2.289211	-2.859843	-0.988488
H	-3.243605	-2.951287	-0.455011
H	-1.697236	-3.739793	-0.711269
C	2.638335	0.238681	-1.443481
H	2.000916	-0.526543	-0.990610
H	3.671612	-0.100449	-1.285730
C	0.393471	-7.730200	-0.089077
H	-0.606889	-8.055873	0.220508

H	1.093667	-8.448736	0.354001
C	-0.031541	5.814430	-1.508617
H	0.474178	6.664031	-1.981136
H	0.693816	4.993198	-1.461806
C	-1.563971	-1.559336	-0.627056
H	-2.207726	-0.707671	-0.869474
H	-0.683830	-1.459297	-1.273833
C	2.433321	1.603845	-0.789443
H	1.372415	1.881140	-0.832355
H	2.975002	2.362567	-1.364452
C	0.691573	-6.301839	0.373173
H	-0.028208	-5.614535	-0.084854
H	1.672509	-6.003186	-0.013125
C	-0.496182	6.188021	-0.088504
H	-1.128214	5.382805	0.301328
H	-1.142006	7.076505	-0.165144
C	2.913469	1.583582	0.667249
H	2.417629	0.764208	1.205758
H	3.985568	1.330927	0.680144
C	-1.130924	-1.504126	0.843913
H	-0.494670	-2.375827	1.056009
H	-2.007293	-1.600679	1.495264
C	0.661289	-6.146200	1.903477
H	1.341235	-6.889537	2.346803
H	-0.339177	-6.381962	2.282214
C	0.636017	6.471139	0.859908
H	1.384303	7.176823	0.497886
C	-0.361689	-0.245992	1.155815
H	0.311259	0.076840	0.363165
C	2.700650	2.880449	1.404405
H	2.471432	3.762138	0.806351

C	1.099401	-4.770623	2.332175
H	2.099735	-4.494200	1.996343
C	0.797443	5.945444	2.079874
H	1.679943	6.245424	2.646351
C	-0.407040	0.503737	2.263469
H	0.238600	1.382333	2.304652
C	2.804362	3.022097	2.730289
H	2.666861	4.016378	3.151167
C	0.414979	-3.866642	3.043551
H	0.905126	-2.913477	3.246808
C	-0.104602	4.947482	2.767083
H	-1.147416	5.086016	2.458704
H	0.179793	3.936357	2.444165
C	-1.233786	0.265624	3.496823
H	-1.849502	1.155125	3.694487
H	-1.929728	-0.568342	3.352750
C	3.119096	1.920491	3.713437
H	4.167801	1.604876	3.600114
H	2.519812	1.028350	3.481694
C	-0.963861	-3.992945	3.630057
H	-1.456741	-4.913197	3.297606
H	-1.580037	-3.162141	3.258590
C	-0.019686	5.008094	4.298734
H	1.032388	4.932654	4.606700
H	-0.369323	5.987407	4.652719
C	2.873883	2.337214	5.168220
H	3.488145	3.219408	5.398302
H	1.830689	2.661390	5.277079
C	-0.370366	-0.010361	4.740862
H	0.227023	-0.915698	4.569365
H	0.351860	0.805719	4.872158

C	-0.958952	-3.940604	5.168347
H	-0.380309	-4.788532	5.557826
H	-0.431425	-3.035618	5.496970
C	-0.816408	3.891750	4.981041
H	-1.873217	3.965807	4.690678
H	-0.465823	2.925893	4.596446
C	3.167122	1.232889	6.186804
H	4.197364	0.876765	6.051353
H	2.517692	0.371582	5.982456
C	-1.195890	-0.167784	6.021127
H	-1.794947	0.739982	6.174327
H	-1.914277	-0.987606	5.889484
C	-2.366567	-3.951681	5.773441
H	-2.889416	-4.865698	5.460659
H	-2.943901	-3.115298	5.355584
C	-0.697663	3.906577	6.507246
H	-1.257426	3.082261	6.961613
H	0.348182	3.810902	6.820750
H	-1.081634	4.843664	6.925752
C	2.969234	1.696255	7.632719
H	3.146752	0.884579	8.345455
H	3.653241	2.515873	7.880320
H	1.949008	2.063521	7.793355
C	-0.337086	-0.429782	7.260427
H	-0.952422	-0.553463	8.157669
H	0.263236	-1.339054	7.141445
H	0.354750	0.398815	7.442222
C	-2.357996	-3.856579	7.301599
H	-3.372966	-3.868002	7.711683
H	-1.808642	-4.694930	7.744708
H	-1.872531	-2.932318	7.633440

Adduct B-C

C	-0.88368	-0.44928	-1.68803
C	-0.58289	-0.75501	-0.33485
C	0.77671	-0.92517	0.04764
C	1.7899	-0.77961	-0.93597
C	1.46901	-0.48241	-2.23484
C	0.11582	-0.31539	-2.61552
H	-1.92299	-0.31385	-1.97377
H	2.82771	-0.90757	-0.64106
H	2.2522	-0.37257	-2.97804
H	-0.12479	-0.07911	-3.64698
C	0.43103	2.43361	1.33603
H	0.08256	3.26229	1.96278
H	1.49662	2.28496	1.53501
H	-0.09571	1.52857	1.65543
C	0.07785	-1.35072	2.33128
H	0.31896	-1.57755	3.36474
C	-1.59525	-0.88637	0.65135
H	-2.63248	-0.75042	0.35735
C	-1.27446	-1.17607	1.9522
C	1.07696	-1.22695	1.40162
H	-2.05699	-1.2714	2.69803
H	2.11657	-1.35567	1.6896
C	0.1732	2.71554	-0.1421
H	0.70897	3.62389	-0.44386
H	0.58511	1.89867	-0.74422
C	-1.31284	2.87206	-0.455
H	-1.74536	3.7195	0.08835

H	-1.48314	3.03426	-1.5237
H	-1.8628	1.97202	-0.15972

Adduct A-C

C	-2.72575	-1.06683	1.04771
C	-1.54971	-1.69823	0.73665
C	-0.30212	-1.13853	1.11626
C	-0.28801	0.08472	1.842
C	-1.52192	0.71307	2.15221
C	-2.71132	0.15537	1.761
H	0.91751	-2.68858	0.2275
H	-3.67344	-1.50171	0.74667
H	-1.55712	-2.63614	0.18789
C	0.93174	-1.75483	0.78316
C	0.95948	0.64813	2.2144
H	-1.50793	1.65077	2.70098
H	-3.6488	0.64793	1.99864
C	2.1356	0.03231	1.87508
C	2.12164	-1.18315	1.15061
H	0.96724	1.58371	2.76684
H	3.08472	0.4755	2.15959
H	3.06001	-1.66073	0.88681
C	-0.36423	1.13845	-1.40628
C	0.96411	1.22406	-1.34951
H	1.4138	1.93612	-0.65932
H	-0.96113	1.77921	-0.75865
C	1.91025	0.36349	-2.14734
H	1.72647	0.50376	-3.22172
H	1.69289	-0.6937	-1.94233
C	-1.12408	0.1711	-2.27527

H	-0.68178	0.14312	-3.27971
H	-1.00249	-0.84231	-1.8667
C	3.37798	0.64854	-1.83689
H	4.04374	0.0125	-2.42708
H	3.62828	1.6922	-2.05352
H	3.58635	0.46957	-0.77715
C	-2.61229	0.49916	-2.36947
H	-2.76831	1.49371	-2.79969
H	-3.1435	-0.22482	-2.994
H	-3.06964	0.48746	-1.37492

Adduct A-D

C	-2.20669	-1.33176	0.21204
C	-1.55838	-1.44828	-1.01551
C	-1.11778	-0.30711	-1.68075
C	-1.32204	0.94968	-1.11679
C	-1.96497	1.06521	0.11208
C	-2.40998	-0.07507	0.77522
H	-2.55279	-2.22148	0.72892
H	-1.39991	-2.42784	-1.45609
H	-0.61197	-0.39862	-2.63704
H	-0.97418	1.83957	-1.63265
H	-2.11212	2.04425	0.55667
H	-2.90986	0.01617	1.7344
C	1.22901	-0.13428	1.0645
H	0.63011	-1.04362	1.09905
C	1.07287	0.7514	2.04819
H	0.35562	0.5166	2.83388
C	2.10825	0.01355	-0.15009

H	3.03916	0.53299	0.1056
H	1.59321	0.65175	-0.8808
C	1.71877	2.10643	2.1376
H	2.65374	2.12739	1.56761
H	1.98284	2.32157	3.17978
C	0.7859	3.2078	1.6155
H	1.24497	4.19684	1.70826
H	0.5454	3.03432	0.56214
H	-0.15683	3.21837	2.17239
C	2.42822	-1.32947	-0.80522
H	1.50461	-1.84104	-1.09586
H	3.04015	-1.20072	-1.70254
H	2.97089	-1.98452	-0.11651

Adduct B-D

C	-1.16635	2.30079	0.49467
H	-1.35155	2.73196	1.48485
H	-1.16423	1.21204	0.59304
H	-2.00669	2.58197	-0.14967
C	0.15354	2.80909	-0.07985
H	0.97869	2.47525	0.56067
H	0.32429	2.34216	-1.05688
C	0.19332	4.32928	-0.2209
H	0.05589	4.81724	0.75019
H	1.146	4.67221	-0.63602
H	-0.60544	4.68199	-0.88249
C	1.23636	-0.56129	0.10198
C	0.59147	-0.58709	-1.13202
C	0.59998	-1.07042	1.23069

C	-0.69009	-1.12067	-1.23769
H	1.0862	-0.18579	-2.011
C	-0.68079	-1.60804	1.12456
H	1.10095	-1.04724	2.19329
C	-1.32583	-1.63304	-0.10925
H	-1.19376	-1.13734	-2.19903
H	-1.17609	-2.00633	2.00453
H	-2.32407	-2.05136	-0.1919
H	2.23226	-0.13757	0.18459
